# Pneumonia—Typhoid Fever—Anæmia:—A Clinic in Bellevue Hospital

**Published:** 1880-11

**Authors:** Austin Flint

**Affiliations:** Professor of Principles and Practice of Medicine in Bellevue Hospital Medical College


ATLANTA
Medical and Surgical Journal.

VOL. XVIII.] NOVEMBER—1880. [No. 8.

### Original Communications.

### PNEUMONIA—TYPHOID FEVER—ANAEMIA:—A CLINIC IN BELLEVUE HOSPITAL.

BY AUSTIN FLINT, M.D.
Professor of Principles and Practice of Medicine in Bellevue Hospital Medical College.

(Reported for the Atlanta Medical and Surgical Journal.)

GENTLEMEN—I shall present to you first a case of pneumonia this morning, which happens apropos. In as much as we have just commenced the consideration of that disease in the didatic course, and this case which I shall present possesses certain points of interest, one of which is the fact that he has really had no treatment, and since he came into the hospital he has convalesced, so that it is a case in which we have the natural history of pneumonia. It is interesting in that point of view.

The expectoration which he has had since he came into the hospital is very characteristic, and I will pass this around. I described it yesterday, a semi-transparent, adhesive, rusty sputa. It presents these characteristics very well.

I will now read the history. The patient's name is Edward P., aged 53 years. I will remark here that the patient's age is always to be considered in the prognosis. After fifty years of age pneumonia involves more danger as a rule than prior to that time. The past history of this patient has no special bearing. He had the initial lesion of syphilis, but that was twenty years ago. Here is a fact, however, to be considered: The patient has been accustomed to the excessive use of alcoholic liquors, and he presented yesterday very distinctly certain symptoms indicative of alcoholism—considerable tremor of the hands, and tongue when protruded.

He entered the hospital on the 19th of October, day before yesterday. He stated that he was quite well up to a week before his admission, when he suddenly became hoarse, and he took cold. For this he took lemon juice and sugar, and in forty-eight hours it had entirely disappeared. He had no chill nor cough at that time. He felt well after that until last Sunday, five days ago. He then was seized with severe, excruciating pain, as he described it, in the right side in the neighborhood of the right nipple. He gives no account of a chill. A well marked chill is pretty constant, indicative of the onset of this disease, but there are exceptions to all pathological rules, and here would seem tc be an exception to that. There was no chill, apparently. The pain, however, was characteristic, the sharp lancinating pain, referred to the region of the nipple on the right side, he described it as sharp in character, and on that account his respirations became accelerated. The severity of the pain was much increased when he took a deep inspiration.

He began about this time to cough, and the cough produced a great deal of pain. The expectoration was transparent, scanty, viscid, and these symptoms continued up to the day of his admission. He was admitted on the 19th, day before yesterday. When attacked last Sunday he was obliged to take to the bed at once.

When admitted he presented an anxious expression, the eyes were injected, the face was flushed, he complained of dispnœa, he had pain in the chest, coughed, complained of debility and loss of appetite; the tongue was coated, and when protruded from the mouth it trembled, showing the effects of alcohol. These symptoms were very apparent yesterday; I find them much better to-day, his expression of face is much improved, there is not the expression of anxiety which he had yesterday, his face is less flushed, there is no appearance of circumscribed flush, which is so distinctive of pneumonia, the tremor is much less than it was yesterday, but that had to do with alcohol.

*Physical signs on admittance.*—Over the lower lobe of the right lung there was dullness on percussion, increased vocal fremitus and bronchophony, and bronchial breathing, and a few crepitant rales. Now, here is a group of physical signs which belongs to the first stage in part, and still more to the early part of the second stage of pneumonia. He was in the second stage of pneumonia when admitted.

On the left side, over a limited area of the lower lobe, all the above enumerated signs were obtained excepting the crepitant rales.

This patient gave, therefore, the evidence afforded by the physical signs of pneumonia, which affected the whole of the lower lobe of the right lung and a limited portion of the lower lobe of the left lung.

*Now the Treatment.*—He had no treatment prior to admission. Well, the only treatment which he received in the hospital was opium, a little to relieve pain, which still persisted to some extent, and as the patient was accustomed to the use of alcoholic stimulants he got three ounces a day of whiskey; this was all the treatment. Why not more? Because there were no indications present. He has had no great amount of pyrexia. Yesterday the temperature was 100.75° at noon. At five o'clock P.M. it was 101.5°, a little increase in temperature, but nothing like hyperpyrexia. This morning the temperature was 100.75°, he rested well all night, and feels much better to-day.

The urine is amber colored, cloudy, throws down a reddish deposit, the urates; has an acid reaction, contains no albumen, and it has the chlorides, as you will see. Here is a little of the urine, and adding a little nitrate of silver you notice it at once throws down a white deposit, and the abundance of this deposit is an evidence that the chlorides are present in considerable quantity in the urine. What is the significance of this? That the deposit in the air cells is not going on, but that resolution is taking place. Probably before this patient was admitted into the hospital there would have been found little, and, perhaps, none of the clorides in the urine. The abundance now, I repeat, shows that there is not any progressive deposit in the lung. But, on the contrary, that the deposit which has taken place is now being absorbed, and we have the physical signs afforded of that fact when we examine the chest.

Now, let us see what are the physical signs at present. First, over the upper lobe on the side upon which pneumonia had extended over tho whole of the lower lobe we get vesiculo tympanitic resonance on percussion ; the percussion sound is more intense, higher in pitch, with tubular and vesicular qualities combined.

Yesterday when I examined this patient I got dullness, marked at the upper part of the lower lobe. Please, gentlemen, always to bear in mind with reference to pneumonia the anatomical situation of the lower lobe. The greater part of that lobe is behind. The interlobular fissure begins about here and takes an oblique course in this way, so that two-thirds of the posterior aspect of the chest are in relation to the lower lobe. Well, up here there was appreciable dullness on percussion, the respiration was broncho-vesicular, well marked bronchophony and bronchial whispering. As I went down the respiration was of a lesser grade broncho-vesicular, and I dropped bronchophony, and got simply increased vocal resonance, and I got subcrepitant rales.

Well, now, taking the history into account the conclusion to be drawn was, that this whole lobe had been solidified, but resolution had begun at the lower part and had progressed further there than up here. The upper part of the lower lobe was still considerably solidified. That was yesterday afternoon. To day the dullness here is somewhat less, the respiration is broncho-vesicular, but there is an increased vesicular quality to it, and bronchophony is now gone, so that we thus have the evidence of considerable progress in resolution since yesterday afternoon. I get to-day also subcreptiant and free crepitant rales, in the stage of resolution we get generally the subcrepitant rale, and not very infrequently we get free crepitant rales, which are distinguished as return crepitant rales, so that we get here a very good illustration of the evidence afforded by physical signs of progressive and rather rapid resolution.

Yesterday I felt a little in doubt whether this portion of the left lower lobe had been solidified, and was undergoing resolution, or whether solidification was just commencing. I can hardly feel the least doubt about it to-day because the solidification is evidently less to-day than it was yesterday. Now, you recollect in the history it was stated that over a limited area we had the same signs that we have on this right side, except the crepitant rales. That space has been marked so that you can see it here. In that space there was a bronchial respiration, bronchophony, bronchophonic whispering. The pneumonia, therefore, had invaded the lower lobe of the left lung. What do I find to-day? I find broncho-vesicular respiration, increased vocal resonance, no bronchophony. The solidification here then, as well as this on the other side, is undergoing rapid resolution.

Please to recollect, while considering the morbid anatomy, that when pneumonia seizes a lobe, in the vast majority of cases it extends over the whole of that lobe; the whole of the lobe becomes solidified. When, however, the second lobe is affected, not infrequently it does not, even when left to itself, without any treatment, extend over the whole of the lobe, but is limited.

You see that line marked in lead pencil. Above that line we get good resonance, and below it we get dullness, but it is not so marked dullness as it was before, because the lower left lobe is in great measure resolved.

Well, gentlemen, this is an interesting case in a certain point of view, to which I have already averted. Here is a patient seized with pneumonia, without a chill, but the other symptoms are such as to leave no question but what he had pneumonia. The treatment none before he entered the hospital, and he has had no potential treatment after he entered the hospital, simply a little palliative treatment and a little sustaining treatment, and the disease you see is running a rapid course from its own intrinsic tendency. We may pronounce this patient convalescent, or nearly so, and this is the fifth day since his pneumonia began. I want to dwell a little on that point because, gentlemen, we are all seekers after truth, of course, and the natural tendency of a disease to end and to recover is always a factor to be taken into account when we would estimate fairly the result of medical treatment. Suppose this patient had come under our observation on the second day, last Monday, being attacked with pneumonia the preceding day; suppose you had bled that patient for example, or given him aconite, or done something else with a view to cut short the disease, or to abridge its duration; how natural it would be for us to say, well here is a case where that method of treatment proved remarkably successful, and yet here you see this has taken place from the inherent tendency of the disease itself to get well. I do not wish to disparage remedial treatment of disease, but it is very desirable that we should learn exactly what it does, and how much is to be attributed to nature.

There is no further indication as regards treatment in this case. The patient is to be kept quiet and comfortable, and unless some complication should occur, and we have no reason to look for any at present, he will very soon be well. He has escaped what there was room to look for, considering his use of alcoholics, and the evidence afforded of their use by his symptoms, the occurrence of delirium tremens. That not infrequently occurs in connection with pneumonia, and forms a grave complication.

*Typhoid Fever.*—The next case, gentlemen, will be one that will interest you. I will state in advance that it is a case of typhoid fever. It will be some time before we reach in the didactic course the consideration of this fever, but bear in mind the facts which the case will present with regard to the temperature, the clinical history, etc.

I call your attention to the congestive redness of the face and to some extent also of the eyes. Observe the hands when I make pressure here, see the white spot which is left, the blood gradually coming into it and reddening it again when pressure is removed. The congestion is more marked on the cheeks than elsewhere, but we have not there a circumscribed redness, such as denotes pneumonia or hectic fever. The redness is gradually shaded off here into a lesser degree, and all over the body, especially the upper part, we have this congestive redness. That is one of the features of this disease. Not peculiar to it, but it is one of the constant features of it.

Observe that although this patient has not an expression of great animation he has not that facies which is so characteristic of typhus and typhoid fever. He has not that expression of indifference, that fatuitous look which when we see it we may almost infer from it that we have a case of one or the other of these two diseases. In typhus, I remark in passing, this congestive redness is still more marked, and gives a dusky hue to the complexion.

His tongue is not much coated. The mental faculties are pretty well preserved; a little blunted, he feels a little dull, but they are pretty well preserved for this fever.

Now let us see what the history says. He is a young man, twenty-one years of age, that is one point; George L. is his name, and he is a butcher by occupation. The patient's habits are good. He says that there was no one ill in the house where he lived. The privy house was in the back yard, and communicated with the one of the next door, but he does not know that any one was ill in the adjoining house. He says that the sinks had a bad odor. Now, these inquiries were made with reference to the cause of the disease. When we take up typhoid fever we shall see that its cause has some connection with fecal excrement, and some hold that it is essential that the dejections from a typhoid patient shall in some way or other find their way into the system, or something derived therefrom find its way into the system, in order for the disease to be produced, while others hold that this something from the typhoid patient is not essential, but that the collection of fecal excrement under certain circumstances produces changes, decomposition results and sometimes from it is received either by drinking water or through the atmosphere into the system that which produces this disease. There is no doubt that the disease can be traced in a large majority of cases to this cause, and hence these inquiries were made. You see there is some ground here in support of that view, but it is not definite.

He was admitted yesterday. I want to say that although a typhoid patient may seem to have the faculties of the mind unaffected, yet we must receive the statement of the patient under these circumstances always with a certain degree of distrust. There is a kind of reluctance in the mind to entertain any thought, and patients who seem to have no aberation of mind whatever will frequently give us statements which are not correct without any intention to deceive, but because they answer at haphazard, as it were. Well, the statements obtained from this patient were, that he had epistaxis a month ago, and this was followed by headache, and loss of appetite, and he also had pains in his limbs and loins, and a general feeling of malaise. This was a month ago, observe, and I make this comment at this point. This is one of the fevers which has a premonitory period, or a prodromatic period of considerable duration. As a rule before the fever becomes developed, before the patient takes to the bed, there have been ailments more or less for at least a week, and not infrequently for a longer period. Now here there seemed to be ailments preceding the development of this fever for nearly a month—three weeks at least.

On Saturday he felt weaker than he felt before, but he still continues to work all day, but on Sunday he felt obliged to take to the bed. So that if we date from Sunday, this is the fifth day. It is very convenient to fix upon some criterion for the commencement of this disease when we take into view the past history of the patient, the history before the patient came under observation, and I know of no better criterion than this taking to the bed. But it is not a perfectly accurate one. I will state to you presently a good reason for supposing that this patient really had typhoid fever for two or three days before he took to bed. He kept about while he still had typhoid fever. But it is the best criterion I know of. Some patients like this will struggle against the disease. They have strength yet and they hope to shake it off by and by, and they go on with their usual occupation. Others, more chicken-hearted, take to bed before the fever is developed. But after all, taking a large number of cases you will find no better criterion for the commencement of the disease than taking to bed.

He took to bed on Sunday, and remained there until removed to the hospital. On Sunday he had slight diarrhœa. In the afternoon he says he had fever and headache which latter continued, but on Monday and Tuesday the diarrhœa did not continue. He came to the hospital, as I have stated, yesterday, Wednesday.

The record for yesterday was this. The face was flushed, as you see it to-day, and the hands were somewhat congested as you see to-day. He had a little appetite ; the tongue was dry, and somewhat coated, of a yellowish color, the coating and color are of no especial importance. Yesterday the temperature was 103.76 deg. He had a good pulse.

He presented the characteristic spots of typhoid fever on the abdomen and back. The abdomen slightly tympanitic. Tenderness and gurgling in the right iliac fossa. In the evening the temperature rose to 104 deg., and that is about the difference between the morning and evening temperature in cases of typhoid fever when there is no disturbing element, such as a complication like pneumonia for example.

The treatment consisted only of a milk diet. No medicines were prescribed ; none were indicated.

This morning the temperature is 102.5 deg., the pulse 88. He rested well through the night, and says he does not feel as tired to-day as he did yesterday.

Well now, let me call your attention to the eruption. It is not abundant, but there is enough of it to show its presence. Now, the eruption of typhoid fever consists of papules, not of spots strictly speaking. It is not a macular eruption, it is a small papular eruption, oval in form, rose colored, the redness disappearing under pressure, returning when the pressure is removed, the eruption consisting frequently of but a few papules, but in some instances we find them quite abundant. They are found on the trunk, the abdomen and chest, anteriorly and posteriorly. We sometimes find them on the extremities. Sometimes they are indeed pretty abundant upon the extremities, never upon the face. These papules are not permanent. They come and go. You see these little marks here; yesterday they were papules, to day they are fading, and to-morrow they will probably have disappeared. There are some papules here which were not apparent yesterday. These are new ones. These will disappear and others will take their place, and so these papules will come and go through the whole course of the disease from now on. I count here upon the abdomen fourteen. There are a good many upon the back also.

What significance pertains to these? A good deal if we take them in connection with other points. They are occasionally found in other diseases. We must recollect that. We must not base a diagnosis upon these papules alone, but if we take them in connection with other points pertaining to the history they do possess great significance, diagnostic significance.

If we limit the term tympanitis to distension of the abdomen we have not it here. If we use the term meteorism to show that we get tympanitic resonance on percussion without distension, we shall probably find that. Yes, everywhere we get tympanic resonance, and if I make pressure here in the right iliac fossa it hurts him. It is not exquisitely tender, but when I make pressure there is an expression of some suffering. If I make pressure in this way I am sensible of a gurgling sound. There is a little tenderness in the left iliac fossa.

¶ Now let us mention here the group of abdominal symptoms which are diagnostic of typhoid fever. The first of all, let me call your attention to one sympton which I read in the history of this patient, but which perhaps did not arrest your attention. Before this fevfr developed he had epistaxis. Now, epistaxis occurs in a very large proportion of cases before the fever is fairly developed, during the prodromic period. It occurs so often that it is of considerable diagnostic importance. It is liable to occur during the course of the fever, but if you are examining the patient in the early part of the fever with reference to the diagnosis you should take into account the occurrence of epistaxis once or repeatedly.

Now, what are the abdominal symptoms, those we can most impress upon the mind. Diarrhoea is one very constant, and yet not absolutely constant. This patient has had enough merely to say that diarrhœa has existed. It does not exist now. He had it one day. The stools when diarrhœa is present are of a yellowish color, ocher color, and this color possesses some importance. Of course diarrhœa alone is not characteristic, it may occur in many affections, but we consider that in connection with other things. There is more or less distension of the abdomen with gas or tympanitis as a rule. This case is an exception to that rule. Tenderness in the iliac fossa, especially on the right side, with gurgling. Now, that constitutes the group of abdominal symptoms. Diarrhœa with yellowish or ocher-colored discharges, more or less meteorism or distension of the abdomen, tenderness more or less all over the abdomen, and especially the iliac fossa, and more particularly in the right iliac fossa, and gurgling on pressure. These are the diognastic abdominal symptoms.

I made the statement that it was certain this patient had typhoid fever before he took to the bed. Now, he took to the bed last Sunday. The eruption does not make its appearance, certainly save in rare exceptions to the rule, prior to the seventh day from the time the patient takes to the bed if he takes to the bed when the fever is first developed. Inasmuch as the eruption was apparent yesterday, which was the fourth day from the time he took to the bed, I am warranted in saying that this patient had the fever upon him for two or three days at least before he took to the bed. I do not know how long the eruption existed before he entered the hospital, but we sometimes meet with cases of this disease which are so mild that the patient does not take to the bed; walking cases they are called. An accident which has to do with the intestinal affection may take place while the patient is walking about, not ill enough to take to the bed; that is, perforation may take place, peritonitis be developed, and it be ascertained autopsically that the patient had typhoid fever. Then although we have here a very mild case we have enough diagnostic points for us to be sure of the diagnosis.

Well now, what shall we do for the patient? Have we any indications? This is the question. We are not to give him remedies as a matter of course, but we are to fulfill the indication. Has he a sufficiently high temperature to call for antipyretic treatment? He gives a temperature of about 104 deg. in the evening; to-day, less. We will see how high it is this evening. If the temperature should rise above 103 deg. except for a very brief period we will place this patient in the cold bath. We will reduce the temperature. That indication may or may not be present. If it should be present we will meet it; if not, the bath will not be called for. What other indications? Are there any disturbances or complications? No. This patient is taking milk as a nourishment. The circulation does not indicate any necessity for alcoholic support, and in truth, except as regards the question of hyperperexia, there is nothing at the present time calling for treatment. There is no diarrhœa to be restricted, there is no restlessness or pain to be palliated by anodynes. But I think it will be a judicious thing to give him some form of the mineral acids which seem to possess some virtues in this disease. We may use dilute hydrochloric or sulphuric acid. Then at the present time that is all we propose to do, to give the patient some one of the mineral acids as a tonic.

*Anæmia.*—I wish to introduce now, gentlemen, another case as a sort of premonition of the subject which I wish to take up on some convenient day, a subject of much interest and importance, and that is anæmia. I consider that so important in its practical relations that for several years, until this year, I have made it a matter of duty to take it up in my first clinical lecture. I can almost always find illustrations of that, and I found that if I did not take it up early in the course, cases illustrating other affections would come up which would take so much time that I was liable to let this have the go-by.

You will notice that there is the anæmic face. There is a pale face, a pale lip, although the excitement of coming before such an audience brings a little hæmatine into the prolabia, yet they are pale; the tongue is rather pale; he is anæmic; there is no question about that; now, we judge of anæmia by the facies, that is to say, very frequently the face shows at once that the blood is deficient in red corpuscles. There has been some progress made of late, gentlemen, with regard to the study of anæmia, and it is now shown that it is not correct that in all cases anæmia consists in a diminished number of the red corpuscles. It has been shown that there may be a diminution of the coloring principle although the red corpuscles are not diminished in number. So we have different kinds of anæmia, and it remains to study further, and ascertain what special relations different forms of anæmia may have, but we will not enter upon that at present.

The face, however, in all cases does not show anæmia, over and over again, I have seen cases where the physical signs, the effect of treatment, etc., showed conclusively that anæmia existed, and yet the face presented no evidence of it. I recollect that some years ago, there was a patient in the hospital, whom I used to bring before this class as an intance of anæmia, and he had one of the richest complexions that you could imagine; a complexion that many women might very well envy, and yet that patient was fairly anæmic, so that we must not trust alone to the appearance of the face.

We may have anæmic or inorganic murmurs at the base of the heart, and so we have here. When we apply the ear or the stethoscope on either side of the heart, over the pulmonary artery or the aorta, we get a systolic murmur. It is quite loud in this case, and it extends even below the base of the heart. If we place the stethoscope upon the neck, to cause the patient to turn the head a little to one side in this way, we get a blowing murmur in the carotid artery, not only that we get what is known as the venous hum. A venous humming sound when you place the stethoscope here. It is sometimes musical; it is not in this instance; it is produced in the veins; you may prove this by placing the finger above tne stethoscope and producing a little pressure there, when you will interrupt the murmur instantly. Remove the pressure and the murmur will instantly return, so that it is not a murmur produced in the artery, as for several years was supposed to be the case. Lannaec observed it I believe, and supposed it to be an arterial murmur, but it is produced in the veins. Is this a reliable sign? I am not prepared to say that it is absolutely so, but it is a highly diagnostic sign. I am not sure we do not find it sometimes in cases where there is no anæmia, but we find it very constantly in anæmic cases. I would make this remark, that the intensity of the murmur does not indicate at all the degree of the anæmia. Thus, we may find it in patients who present all other evidences of not a great degree of anæmia, and again, it is very slight in some who are, judging from the face and other circumstances, highly anæmic. But this sound possesses great practical value in this point of view. If we have a patient under treatment with anæmia, the disappearance of the sign is very reliable evidence that the anæmia has disappeared. If, as not infrequently they do, patients under treatment say they are well, they appear to be well, and we still hear this humming murmur, we should say that in this case there is not that complete recovery which may be expected, and the treatment should be continued until the hum disappears.

I shall content myself, then, to-day gentlemen, with calling your attention to the anæmic facies, and to the facts which I have stated with regard to the physical signs, I will say, in conclusion, that the anæmia in this case had been produced by intermittent fever, malaria, and had I time I would call your attention to that subject. I open up this subject of anæmia because it meets us on all hands in practice, and it is very essential that it be taken into view in practice not only in cases where that is the only malady to be treated, but in a large number of cases in which it is an element connected with other affections.

